# Telomere Lengths, Pulmonary Fibrosis and Telomerase (*TERT*) Mutations

**DOI:** 10.1371/journal.pone.0010680

**Published:** 2010-05-19

**Authors:** Alberto Diaz de Leon, Jennifer T. Cronkhite, Anna-Luise A. Katzenstein, J. David Godwin, Ganesh Raghu, Craig S. Glazer, Randall L. Rosenblatt, Carlos E. Girod, Edward R. Garrity, Chao Xing, Christine Kim Garcia

**Affiliations:** 1 McDermott Center for Human Growth and Development, University of Texas Southwestern Medical Center, Dallas, Texas, United States of America; 2 Division of Pulmonary and Critical Care Medicine, Department of Internal Medicine, University of Texas Southwestern Medical Center, Dallas, Texas, United States of America; 3 Department of Pathology, The State University of New York Upstate Medical University, Syracuse, New York, United States of America; 4 Department of Radiology, University of Washington Medical Center, Seattle, Washington, United States of America; 5 Division of Pulmonary and Critical Care Medicine, Department of Internal Medicine, University of Washington Medical Center, Seattle, Washington, United States of America; 6 Division of Pulmonary and Critical Care Medicine, Department of Internal Medicine, University of Chicago, Chicago, Illinois, United States of America; University of Giessen Lung Center, Germany

## Abstract

**Background:**

Telomerase is an enzyme that catalyzes the addition of nucleotides on the ends of chromosomes. Rare loss of function mutations in the gene that encodes the protein component of telomerase (*TERT*) have been described in patients with idiopathic pulmonary fibrosis (IPF). Here we examine the telomere lengths and pulmonary fibrosis phenotype seen in multiple kindreds with heterozygous *TERT* mutations.

**Methods and Findings:**

We have identified 134 individuals with heterozygous *TERT* mutations from 21 unrelated families. Available medical records, surgical lung biopsies and radiographs were evaluated retrospectively. Genomic DNA isolated from circulating leukocytes has been used to measure telomere lengths with a quantitative PCR assay. We find that telomere lengths of *TERT* mutation carriers decrease in an age-dependent manner and show progressive shortening with successive generations of mutation inheritance. Family members without *TERT* mutations have a shorter mean telomere length than normal, demonstrating epigenetic inheritance of shortened telomere lengths in the absence of an inherited *TERT* mutation. Pulmonary fibrosis is an age-dependent phenotype not seen in mutation carriers less than 40 years of age but found in 60% of men 60 years or older; its development is associated with environmental exposures including cigarette smoking. A radiographic CT pattern of usual interstitial pneumonia (UIP), which is consistent with a diagnosis of IPF, is seen in 74% of cases and a pathologic pattern of UIP is seen in 86% of surgical lung biopsies. Pulmonary fibrosis associated with *TERT* mutations is progressive and lethal with a mean survival of 3 years after diagnosis. Overall, *TERT* mutation carriers demonstrate reduced life expectancy, with a mean age of death of 58 and 67 years for males and females, respectively.

**Conclusions:**

A subset of pulmonary fibrosis, like dyskeratosis congenita, bone marrow failure, and liver disease, represents a “telomeropathy” caused by germline mutations in telomerase and characterized by short telomere lengths. Family members within kindreds who do not inherit the *TERT* mutation have shorter telomere lengths than controls, demonstrating epigenetic inheritance of a shortened parental telomere length set-point.

## Introduction

Telomerase is a multimeric ribonucleoprotein enzyme that catalyzes the addition of repetitive DNA sequence to telomeres, specialized structures at the ends of chromosomes. The human enzyme consists of both a functional RNA (hTR) and a reverse transcriptase protein component (hTERT)[1], [2]. Its action on telomeres solves the end-replication problem by counteracting the progressive shortening of the chromosome that occurs with each cell division. hTERT is highly expressed in germ cells, cells with proliferative potential and immortalized cancer cells[3], [4], [5].

Telomerase activity is restricted in humans. Evidence of this is seen by the progressive shortening of telomere lengths of human mononuclear leukocytes with age[6] and several diseases of inherited telomerase dysfunction[7]. Mutations in the genes encoding the telomerase complex were first found in patients with dyskeratosis congenita (DKC), and then in patients with bone marrow failure syndromes, pulmonary fibrosis and liver disease. While these are very different clinical diseases, a subset of each share a common pathogenesis related to short telomere lengths due to inherited germline telomerase mutations. DKC is a very rare multisystem disorder (prevalence of 1 in one million individuals) affecting children. Most DKC patients have mutations in the X-linked *DKC1* gene, which encodes dyskerin[8]; fewer patients have mutations in *TERC*, which encodes hTR[9]. Case reports of rare kindreds have been described with mutations in *TERT* [*10*] *, NOP10* [*11*] *, NHP2* [*12*] *and TINF2* [*13*], which encode different components of the telomerase and the telomere complex. DKC is considered a syndrome of premature aging with death occurring at a median age of 16 years and a maximum of 50, usually from bone marrow failure, cancer or pulmonary disease[14].

Like DKC, idiopathic pulmonary fibrosis (IPF) is a lethal disease; patients have a median survival of approximately 3–5 years after diagnosis[15]. However, unlike DKC, IPF is much more common with its prevalence increasing with age to 65 cases per 100,000 for those 75 years or older [16]. Heterozygous loss-of-function mutations in *TERT* have been found in up to 15% of kindreds with the familial pulmonary fibrosis[17], [18] and in 1–3% of sporadic cases[19], [20]. IPF is one of the most common interstitial lung diseases (ILDs), a heterogeneous collection of lung disorders affecting mainly the supporting structures (interstitium) of the lung. A diagnosis of IPF portends a much worse prognosis than most other ILDs and predicts the lack of a therapeutic response with prednisone, which is commonly used to treat other ILDs[15]. Within our initial reports of heterozygous *TERT* mutation carriers, most affected with pulmonary fibrosis were diagnosed with IPF but some had a granulomatous lung disease or pulmonary fibrosis which was not consistent with IPF. This raises the question of the actual spectrum of pulmonary phenotypes associated with *TERT* mutations.

Using probands initially identified with heterozygous *TERT* mutations and pulmonary fibrosis, we have expanded these families to identify 134 carriers of heterozygous *TERT* mutations, ranging in age from 5 to 88, with a mean age of 51 years. We have collected information regarding their medical history and environmental and occupational exposures. Here we show that 40% of *TERT* mutation carriers with a mean age of 51 years have self-reported pulmonary fibrosis, which is usually but not always clinically consistent with IPF. The relative frequencies of bone marrow dysplasias, cancer and other diseases are described for this cohort. Telomere lengths of mutation carriers in successive generations of the three largest kindreds show progressive telomere shortening. Related family members who do not inherit the *TERT* mutation have shorter telomeres than controls, demonstrating epigenetic inheritance of a shortened parental telomere set-point.

## Methods

### Human Subjects

This study was approved by the University of Texas Southwestern Medical Center Institutional Review Board. Cohorts of familial pulmonary fibrosis kindreds and sporadic cases of idiopathic interstitial pneumonias were collected as described[19]. Each sporadic case and at least one member of each kindred with familial pulmonary fibrosis carried a diagnosis of IPF or unclassifiable interstitial pneumonia in concordance to established criteria[15]. Written informed consent was obtained from all subjects. Each participant completed a questionnaire and self-reported their ethnicity, medical history, pulmonary symptoms, and exposures to fibrogenic medications, radiation and particulates. Medical records, open lung biopsy samples and radiographic studies were obtained when available. Genomic DNA was isolated from circulating leukocytes with an Autopure LS (Qiagen, Valencia, CA) or from formalin-fixed paraffin-embedded archived tissue using the QIAmp DNA Mini Kit (Qiagen). Genomic DNA samples for the population of normal control subjects (n = 195, age 19–89 years) were obtained from a cohort of unrelated, multiethnic individuals from Dallas, Texas kindly provided by H. H. Hobbs.

### CT Chest Scan Analysis

Available CT scans of the chest were obtained from different US medical centers and were independently reviewed by a chest radiologist (J.D.G.). Each scan was categorized as “typical or UIP” as defined in [15] with peripheral and basal reticulations, traction bronchiectasis and honeycombing. Those deemed “consistent with UIP” lacked honeycombing.

### Surgical Lung Biopsy Analysis

Available lung biopsies were independently reviewed by a pulmonary pathologist (A.-L.K.).

### Sequencing and Mutation Analysis

Sequencing of both *TERT* and *TERC* were performed as described[18].

### Determination of Telomere Length

Genomic DNA was quantitated in triplicate using a ND-8000 spectrophotometer (NanoDrop Technologies; Wilmington, DE), stored at 50 ng/µl in TE (10 mM Tris, pH 8, 1 mM EDTA), diluted to 2 ng/µl in water and added as a 20 ng aliquot to the final reaction. Multiplexed quantitative polymerase chain reaction (PCR) determination of telomere lengths were performed on a RotorGene real-time PCR system (Qiagen) as described[21] with 0.15X SYBR Green I (Invitrogen) and the single copy Albumin gene primers at a final concentration of 500 nM. A standard curve was generated using serially diluted genomic DNA (ranging from 5 to 40 ng) pooled from 5 control individuals. Reference DNA isolated from cell line MCF7 (ATCC Cat#HTB-22), which has very short telomeres, and two control individuals were included on each run. RotorGene Q Software version 1.7.94 was used for the construction of standard curves and crossing point (Ct) values. T/S ratios were calculated as previously described[19]. Each sample was assayed in triplicate. The final reported T/S value is the average T/S from two independent experiments. The relative T/S ratio was calculated by dividing the sample T/S value by the T/S value of the reference MCF7.

### Telomerase Repeat Amplification Protocol (TRAP) Assay

Missense mutations in the *TERT* gene were introduced into the parental plasmid pGRN125 and TRAP assays were performed as described[19].

### Statistical Methods

Analyses on quantitative traits were performed by fitting linear models and contingency tables were tested by using Fisher's exact test. All analyses were performed with the R software package, version 2.8.1 (www.r-project.org). The log transformed relative T/S ratio was normally distributed.

## Results

**Figure 1:**
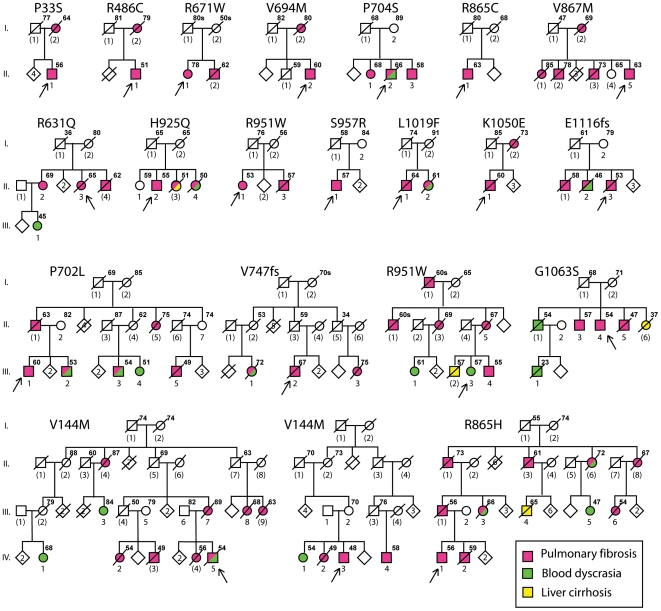
Abridged pedigrees of kindreds with heterozygous mutations in the gene encoding the protein component of telomerase (*TERT*). The arrow indicates the index case. Circles represent females; squares represent males. Symbols with a slash indicate deceased subjects. Individuals with pulmonary fibrosis, blood dyscrasias and liver cirrhosis are indicated by red, green, and yellow symbols, respectively. Roman numerals indicate the generation. Numbers in parentheses indicate individuals for whom no DNA sample was available. The age at the time of consent or the age of death is indicated to the upper right of each symbol. The predicted amino acid changes that result from the *TERT* mutations are listed above each family. Some of the pedigrees have been modified to hide identifying features.

We have identified 21 different families with heterozygous coding mutations in the gene encoding the protein component of telomerase, *TERT* (**Figure 1** **and Table S1**). Most of the families have been previously reported[18], [19]. Eight have been identified more recently and all have been more fully expanded. From our collection of 106 unrelated kindreds with familial pulmonary fibrosis, 19 (18%) have been found with heterozygous *TERT* mutations. Previously, we reported that in a group of unrelated individuals with an idiopathic interstitial pneumonia and no family history of pulmonary fibrosis in a first or second-degree family member, 2 (3%) had a *TERT* mutation. We have also expanded these two families (S957R and R865C in **Figure 1**) and included them in the analysis. All of the missense mutations involve conserved residues in regions of the protein that have postulated roles in enzyme activity; none of the mutations were reported in a multiethnic panel of 528 controls[22] or are present in the SNP database. Two of the mutations (V144M and R951W) have been found in different unrelated families[18]. All the new mutations (R631Q, R671W, V867M, H925Q, R951W and G1063S) have not been identified previously and demonstrate reduced *in vitro* telomerase activity as measured by the telomere repeat amplication protocol (TRAP) assay (**Figure S1**). All probands have been sequenced for the entire coding exons and flanking introns of *TERT* and *TERC*. Only the families with *TERT* mutations have been included in this analysis.

Direct genomic DNA sequencing for the proband's *TERT* mutation in the expanded families identified 134 heterozygous *TERT* mutation carriers with a mean age of 51 years. For some deceased individuals, archived formalin-fixed paraffin embedded tissue was obtained and directly sequenced for the mutation. Only individuals for whom the mutation was directly sequenced or could be inferred based upon the family structure have been included in our analysis. Most of the mutation carriers come from three large pedigrees (**Figure 1**). The self-described ethnicity of 90% of mutation carriers is white, the remainder is Hispanic.

Each subject completed a medical questionnaire including self-reporting of medical diagnoses; these were confirmed with medical records when available. Forty percent of the mutation carriers are affected with pulmonary fibrosis and almost half reported one of the following respiratory diseases: pulmonary fibrosis, obstructive sleep apnea, chronic obstructive pulmonary disease, asthma or previous pneumothorax. Gastroesophageal reflux disease is reported by 26%, with gastritis and/or peptic ulcer disease found in ∼8%. Eight percent of patients have elevated liver function tests and for one of these, cryptogenic liver cirrhosis was diagnosed. Osteopenia and/or osteoporosis are reported by over one-fourth of mutation carriers. Over 15% have at least one blood dyscrasia; mild anemia is more common than aplastic anemia or myelodysplastic syndrome combined. The relative frequencies of other diseases are listed in **Table 1**.

**Table 1:** Phenotype of 134 subjects with heterozygous *TERT* mutations.

		Number of Subjects	Percent (%)
**Age**			
Mean	51		
Median	54		
Range	5–88		
**Ethnicity, Total**			
Caucasian		119	89.6
Hispanic		15	11.2
**Respiratory/Pulmonary Disease, Total**		**65**	**48.5**
Pulmonary Fibrosis		53	39.6
Obstructive Sleep Apnea		11	8.2
Asthma		8	6.0
COPD		4	3.0
Pneumothorax		2	1.5
**Gastrointestinal Disease, Total**		**48**	**35.8**
GERD		35	26.1
Gastritis/PUD		11	8.2
Elevated LFTs		11	8.2
**Musculoskeletal Disease, Total**		**40**	**29.9**
Osteopenia/Osteoporosis		35	26.1
Scoliosis		4	3.0
**Blood Dysrasias, Total**		**22**	**16.4**
Anemia		18	13.4
Aplastic Anemia/MDS		4	3.0
**Psychiatric Disease, Total**		**18**	**13.4**
Depression		15	11.2
Anxiety		6	4.5
**Endocrine Disease, Total**		**18**	**13.4**
Hypothyroidism		13	9.7
Diabetes Mellitus		5	3.7
**Cancer, Total**		**14**	**10.5**
Lung		3	2.2
Breast		3	2.2
Stomach		2	1.5
Lymphoma		2	1.5
Others (Endometrial, Skin)		4	3.0
**Cardiovascular Disease, Total**		**7**	**5.2**
Atherosclerosis†		6	4.5
Cardiac Valvular Disease		2	1.5
**Neurologic Disease, Total**		**5**	**3.7**
Mental Retardation		2	1.5
Seizures		2	1.5
**Other**			
Recurrent Infections‡		6	4.5
Corneal Dystrophy		2	1.5

COPD, chronic obstructive pulmonary disease; LFTs, liver function tests; MDS, myelodysplastic syndrome; CAD, coronary artery disease.

†Atherosclerosis includes those subjects with coronary arterial disease, peripheral vascular disease, stroke and transient ischemic attacks.

‡Recurrent pulmonary, cutaneous, bone and/or renal infections.

**Figure 2:**
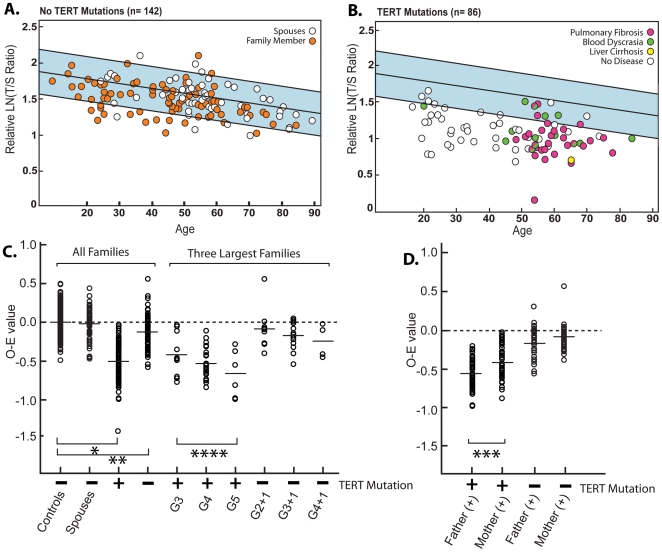
Telomere lengths of family members from *TERT* kindreds. Mean telomere lengths as measured by a quantitative PCR assay for (**A**) normal subjects and (**B**) subjects with *TERT* mutations are plotted against age. (**A**) The telomere lengths of spouses (open symbols) and family members without *TERT* mutations (orange filled symbols) are shown relative to the 50^th^ percentile (center line) and the 10^th^ to 90^th^ percentiles for 195 unrelated healthy individuals from 19–89 years of age (blue shaded region). (**B**) *TERT* mutation carriers without any clinical disease (open circles), with pulmonary fibrosis (red symbols), blood dyscrasias (green symbols) and liver cirrhosis (yellow symbols) are plotted against the same reference range. (**C**) Mean observed minus expected age-adjusted telomere length for the indicated groups. The minimum number of successive generations the *TERT* mutation has segregated in the kindred is indicated; G3 indicates subjects in the third successive generation with *TERT* mutations, i.e., the children with *TERT* mutations whose parents and grandparents also had a *TERT* mutation. G2+1 indicates subjects who do not have a telomerase mutation and are the offspring of individuals that represent the second successive generation with a *TERT* mutation. Bars show the mean value. (**D**) Mean observed minus expected age-adjusted telomere lengths for offspring of *TERT* mutation carriers with (+) and without (−) the mutation. Mean telomere lengths are shorter for offspring of fathers who carry a *TERT* mutation. P-values of 2.4×10^−38^ (*),1.01×10^−5^ (**), 0.01 (***) and linear trend test P-value of 0.04 (****).

Telomere length of genomic DNA isolated from circulating leukocytes was determined using a modified multiplexed quantitative PCR assay. In short, this assay assesses the telomere length as a ratio of the telomere copy repeats to a single copy gene, relative to a reference sample. We have observed good correlation between this method and the standard Southern-blot method (terminal restriction fragment length analysis) for determining telomere length (Spearman's rank correlation = 0.83, P-value <2.2×10^−16^, **Figure S2**). We found that those with a *TERT* mutation (n = 86) have significantly shorter telomere lengths than an unrelated healthy cohort ranging in age from 19–89 years of age (P-value  = 2.4×10^−38^, **Figure 2B, 2C**). Approximately 80% of *TERT* mutation carriers fell below the 10^th^ percentile of the reference group; all were shorter than the 50^th^ percentile (**Figure 2B**). Most of the *TERT* mutation carriers with pulmonary fibrosis, blood dyscrasias or liver cirrhosis were 40 years of age or older. The age-related decline in telomere lengths of the *TERT* mutation carriers was similar to that of the normal controls.

When comparing telomere lengths between individuals, the values are age-adjusted and reported as an observed minus expected value (O-E value) (**Figure 2C, 2D**). There was no difference between telomere lengths of the reference population and the unrelated spouses, but we find that the mean telomere length of related family members without a *TERT* mutation was significantly shorter than the reference population (P-value  = 1.01×10^−5^). For the three largest kindreds, we were able to estimate a minimal number of generations through which the *TERT* mutation segregated. For these three families, the mean telomere length of the *TERT* mutation carriers progressively shortened for each *TERT*-carrying generation, G3 through G5 (**Figure 2C**) (Linear trend test P-value  = 0.04). The spread of telomere lengths of *TERT* mutation carriers was sufficiently broad to partially overlap the spectrum of lengths seen in the normal controls, spouses and related family members. We also studied the telomere lengths of children of *TERT* mutation carriers that did not inherit their grandparents' and parents' mutation (G2+1) and compared these to age-matched controls. Although not statistically significant, there was again a trend toward shorter telomere lengths of children without a *TERT* mutation who were born to *TERT* carriers corresponding to the number of successive generations the mutation segregated in the family. We investigated the telomere lengths as related by genotype and parental gender (**Figure 2D**). The telomere lengths of children who inherited a *TERT* mutation were shorter if the mutant allele was transmitted from the father rather than the mother, even after adjusting for the sex of the child (P-value  = 0.01). A *TERT* mutation not transmitted from parent to child led to an increase in telomere length, regardless of parental gender, that did not reach the mean telomere length of controls.

Since pulmonary fibrosis is common in *TERT* mutation carriers, we sought to more carefully characterize the clinical interstitial lung disease phenotype (**Table 2**). We obtained and independently reviewed all available medical records and archived radiographic and pathologic specimens for those with a self-reported or family-reported diagnosis of pulmonary fibrosis. Only older adults were affected, with diagnoses made between 42 to 83 years of age. In general, men were more commonly affected (60%) than women (40%) and had an earlier clinical presentation, with a mean age of 54 vs. 63 years, respectively. Dyspnea and crackles were almost uniformly seen.

**Table 2:** Features of 53 *TERT* mutation carriers with pulmonary fibrosis.

Feature		Number of Subjects	Percent (%)
**Age, years**			
Range	42–83		
Mean	57		
Mean, Men	54		
Mean, Women	63		
**Gender**			
Male		32	60
Female		21	40
**Symptoms and Signs (n = 51)**			
Dyspnea		51	100
Crackles		49	96
Cough		44	86
Clubbing		21	41
**Smoking (n = 49)**			
Former/Current		31	63
Never		18	37
**Pack-Years of Cigarette Smoking (n = 22)**			
Mean	21		
Range	2–75		
**Self-reported Fibrogenic Exposure (n = 28)** *			
With Exposure		20	71
Without Exposure		8	29
**Smoking and/or Fibrogenic Exposure (n = 28)** *			
With Exposure		27	96
Without Exposure		1	4
**Pattern by CT scans (n = 39)**			
Typical of UIP		29	74
Consistent with UIP except for the absence of honeycombing		5	13
Not typical for UIP		5	13
Enlarged mediastinal lymph nodes		15	38
Superimposed emphysema		8	20
**Expiratory High Resolution CT scans (n = 16)**			
Air-trapping		6	38
No air-trapping		10	62
**Surgical lung specimens (n = 29)**			
UIP		25	86
Not classifiable		3	10
Insufficient tissue		1	3

*A fibrogenic exposure was noted if the subject self-reported a drug, radiation, occupational or environmental exposures that have been clinically linked to the development of an interstitial lung disease in a completed pulmonary questionnaire.

Pulmonary function tests and diffusion capacity measurements were available for a subset of those with pulmonary fibrosis (**Table 3**). All affected individuals had a decrease in the diffusion capacity, a cardinal parameter of IPF. In addition, the majority had evidence of restrictive physiology.

**Table 3:** Pulmonary function tests of *TERT* mutation carriers with pulmonary fibrosis.

	Mean ± SD	Percent Predicted (%)	Percent of Subjects (%)
**FVC (L) (n = 46)**	2.14±0.91	55±27	
Range	(0.91–4.30)	(27–127)	
**FEV1 (L) (n = 46)**	1.77±0.69	60±18	
Range	(0.74–3.42)	(25–111)	
**Ratio of FEV1/FVC (n = 46)**	87±11		
Range	(50–126)		
**TLC (n = 35)**	3.59±0.93	60±13	
Range	(2.01–5.97)	(38–90)	
**DL_CO_ (n = 38)**	9.2±3.1	37±11	
Range	(3.3–16.0)	(19–58)	
**PFT Pattern (n = 47)** †			
Normal			4
Restrictive			87
Obstructive			0
Mixed			9
**Diffusion Capacity (n = 38)**			
DLco <75% predicted			100

FVC, Forced vital capacity; FEV1, Forced expiratory volume in one second, TLC, total lung capacity; DL_CO_, Diffusion capacity for carbon monoxide.

† An obstructive pattern is indicated by a FEV1/FVC ratio <75%. A restrictive pattern is indicated either by a total lung capacity of ≤75% or by a FEV1/FVC ratio of ≥75% with a FVC <80%. A mixed pattern is indicated by evidence of restriction and obstruction.

Over half were former or current smokers with a mean 21 pack-year cigarette smoking history. Each of the living *TERT* mutation carriers completed a pulmonary questionnaire that included self-reported drug, radiation, occupational or environmental exposures that have been linked to the development of pulmonary fibrosis. Over ninety-five percent of *TERT* mutation carriers with pulmonary fibrosis report an exposure to smoking and/or a fibrogenic environmental or occupational agent that may have contributed to the development of their interstitial lung disease. There appears to be a significant association between smoking and/or fibrogenic exposures with pulmonary fibrosis in *TERT* mutation carriers who are ≥40 years of age. (**Table 4**).

**Table 4:** Relationship between smoking, fibrogenic exposures and pulmonary fibrosis in *TERT* mutation carriers ≥40 years of age.

Exposure	No. of Subjects with Exposure	No. of Subjects without Exposure	P-Value*	Odds Ratio [95% confidence interval]
**Smoking, present or past**				
Pulmonary Fibrosis	20	8	0.02	4.0 [1.2, 14.5]
No Pulmonary Fibrosis	11	18		
**Fibrogenic Exposure** **				
Pulmonary Fibrosis	20	8	0.18	2.3 [0.7, 8.1]
No Pulmonary Fibrosis	15	14		
**Smoking and/or Fibrogenic Exposure**				
Pulmonary Fibrosis	27	1	0.005	13.6 [1.7, 636.8]
No Pulmonary Fibrosis	19	10		

*By Fisher's exact test.

**The self-reported fibrogenic exposures include ingestion of methotrexate and nitrofurantoin; exposure to birds and bird antigens including parakeets, cockatiels, and eagle feathers; occupational exposures to asbestos, welding, carpentry, mining, sandblasting, cement manufacturing, railroad work, insulation; and household exposure to water damage and significant mold.

**Figure 3:**
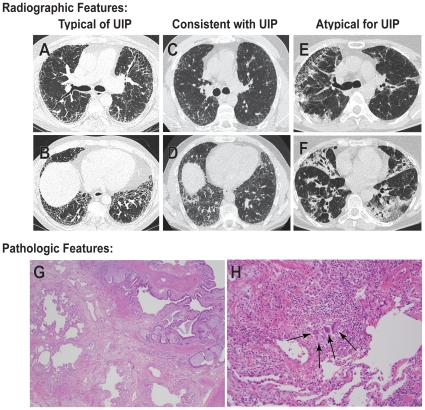
Radiographic and pathologic findings of heterozygous *TERT* mutation carriers with pulmonary fibrosis. Computed tomography (CT) scans of three different subjects with pulmonary fibrosis. Representative cases are shown with a pattern typical of Usual Interstitial Pneumonia (UIP) with peripheral, basal-predominant fibrosis and moderate to severe honeycombing (**A,B**), a pattern consistent with UIP with peripheral, basal-predominant fibrosis in the absence of honeycombing (**C,D**), and a pattern atypical for UIP with fibrosis predominantly affecting the upper lobes and along the bronchi (**E, F**). Scans are shown at the level of the carina (**A, C, E**) and the lung base (**B, D, F**). The majority (25 cases or 86%) of *TERT* mutation carriers with lung specimens available for review had diagnostic histologic features of UIP. In this low magnification view of UIP (**G**), typical variegated honeycomb areas (top right) are seen alternating with normal areas (left) and scarred lung (bottom). The case shown in (**H**) shows increased inflammation and a small, loosely aggregated non-necrotizing granuloma (arrows) that is characterized by a cluster of epitheloid histiocytes and multinucleated giant cells surrounded by chronic inflammation in the interstitium. Panels G and H are shown at 40 and 100-fold magnification, respectively.

Radiographs, including CT scans of the chest, were evaluated for 39 different cases. For 29 (74%) subjects, the pattern of pulmonary fibrosis was typical for UIP, that is, there was patchy reticulation concentrated in the periphery and bases which was accompanied by honeycombing (**Table 2**). Honeycombing was generally mild or moderate, but occasionally severe. For 5 subjects, the pattern of fibrosis was consistent with UIP except for an absence of honeycombing. For the remaining 5 subjects, the CT scans were atypical of UIP because the fibrosis was predominantly located in the mid or upper lung fields or along the bronchi. **Figure 3** shows representative CT scans typical of UIP, consistent with UIP but without honeycombing, and atypical for UIP. Enlarged mediastinal lymph nodes (exceeding 1 cm in the short axis) were found in 15 (38%) cases. Sixteen cases included expiratory scans, which detected air-trapping in 6 cases. All cases with a radiographic pattern typical or consistent with UIP had a pathologic diagnosis of UIP (n = 21; **Table 5**). Regardless of the specific radiographic pattern, all subjects with serial CT radiographs (n = 19) showed progression.

**Table 5:** Clinical diagnosis, radiographic pattern and pathologic features of 29 *TERT* mutation carriers with surgical lung specimens.

*TERT* Mutation	Clinical Diagnosis	Radiographic Pattern*	Pathologic Pattern†	Additional Pathologic Features
P33S	IPF	UIP	UIP	
V144M	IPF	Consistent with UIP	UIP	
V144M	IPF	Not available	UIP	
V144M	IPF	Not available	UIP	
V144M	chronic HP	Atypical for UIP	UIP	Increased inflammation, BOOP
V144M	chronic HP	UIP	UIP	Increased inflammation, scattered granulomas
R486C	IPF	Consistent with UIP	UIP	
R631Q	IPF	UIP	UIP	DAD
V694M	IPF	UIP	UIP	
P702L	IPF	UIP	UIP	
P702L	IPF	UIP	UIP	
P702L	IPF	Not available	UIP	Increased inflammation, scattered granulomas
P702L	IPF	UIP	UIP	Increased inflammation, NSIP-like areas
P704S	IPF	UIP	UIP	Increased inflammation, NSIP-like areas
V747fs	IPF	UIP	UIP	Increased inflammation
R865H	IPF	UIP	UIP	Increased inflammation, rare granulomas
R865H	IPF	Consistent with UIP	UIP	Increased inflammation, rare granulomas
R865H	Interstitial pneumonitis	Atypical for UIP	Not classifiable	DAD and honeycombing
R865H	Unclassifiable pulmonary fibrosis	Not available	Not classifiable	BOOP and subpleural fibrosis
R865C	IPF	Atypical for UIP	UIP	Increased inflammation, rare granulomas
H925Q	IPF	UIP	UIP	
H925Q	IPF	UIP	UIP	
R951W	IPF	UIP	UIP	
R951W	IPF	UIP	Insufficient tissue for diagnosis	Fragment of subpleural scar
L1019F	IPF	Consistent with UIP	UIP	BOOP
K1050E	IPF	UIP	UIP	Increased inflammation
G1063S	IPF	UIP	UIP	
G1063S	IPF	UIP	Not classifiable	Chronic interstitial pneumonia with fibrosis
E1116fs	IPF	Not available	UIP	BOOP

UIP, usual interstitial pneumonia; HP, hypersensitivity pneumonitis; BOOP, bronchiolitis obliterans organizing pneumonia; NSIP, nonspecific interstitial pneumonia; DAD, diffuse alveolar damage.

*CT scans of the chest were categorized based upon the pattern of pulmonary fibrosis (see text).

†Lung specimens with histologic features of “UIP” had a heterogenous mixture of interstitial fibrosis containing collagen deposition and fibroblast foci, islands of normal lung, and areas of architectural distortion with parenchymal scarring and honeycomb change.

Lung biopsy specimens from 29 cases, including 22 surgical biopsies and 8 explants (both biopsy and explant were available for one case) were reviewed independently. The majority (25 cases or 86%) had diagnostic histologic features of UIP with a characteristic heterogeneous mixture of interstitial fibrosis containing both collagen deposition and fibroblast foci, islands of normal lung, and areas of architectural distortion with parenchymal scarring and/or honeycomb change. In 10 cases (35%) chronic inflammation, consisting of a mixture of lymphocytes and plasma cells, was increased in the scarred areas of the lung and in adjacent interstitium compared to that usually seen in typical UIP. Another unusual feature seen in 5 cases (17%) was the presence of scattered histiocytes and/or small, loose non-necrotizing granulomas within the interstitium (**Table 5**); these are also not usually seen in typical UIP. Areas of acute lung injury, including bronchiolitis obliterans-organizing pneumonia (BOOP) or diffuse alveolar damage (DAD) were superimposed on UIP in 4 cases, and were indicative of the accelerated form of UIP/IPF. Four cases could not be classified as UIP. The diagnosis in one could not be established because of the extent of DAD superimposed on the honeycomb change. Clinically, this subject was diagnosed with interstial pneumonitis and died less than 8 weeks from the start of her symptoms (**Table 5**). One case showed only BOOP along with unclassifiable subpleural fibrosis; this subject died from respiratory failure secondary to “COPD and pulmonary fibrosis” four years from diagnosis. Another case had chronic interstitial pneumonia with fibrosis that could not be further classified; this subject died 14 months after the surgical lung biopsy was obtained. A final case contained only a minute fragment of subpleural scar that was considered insufficient for diagnosis. For all four non-classifiable cases, the biopsies were taken from a single lobe.

**Figure 4:**
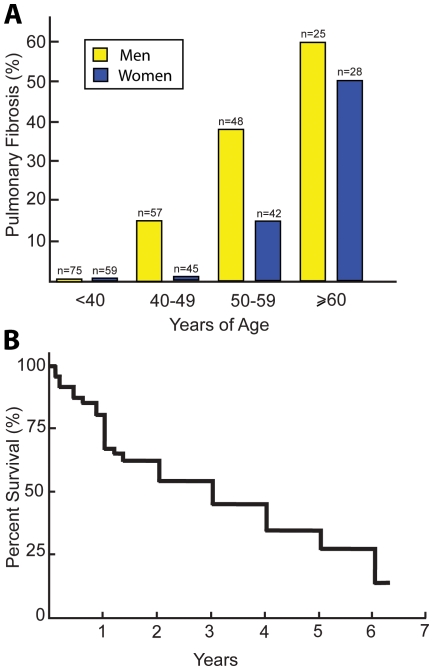
Pulmonary fibrosis is a lethal, age-associated phenotype of *TERT* mutation carriers. (**A**) Penetrance of pulmonary fibrosis is shown for men (yellow) and women (blue bars) of different ages. No one less than 40 years of age exhibited pulmonary fibrosis. Penetrance of pulmonary fibrosis for men vs. women 40–49, 50–59 and ≥60 years of age is 14% vs. 2%, 38% vs. 14%, and 60% vs. 50%, respectively. (**B**) Kaplan-Meier survival curve of 47 different *TERT* mutation carriers with pulmonary fibrosis demonstrate a mean survival of 3 years after diagnosis.

Pulmonary fibrosis for *TERT* mutation carriers is an age-related phenotype. None of the mutation carriers were diagnosed with pulmonary fibrosis prior to 40 years of age. The penetrance of pulmonary fibrosis increased to 60% and 50% for men and women, respectively, ≥60 years of age (**Figure 4A**). Individuals heterozygous for *TERT* mutations died at an early age. For 29 male mutation carriers, the average age of death was 57.7. For 24 female mutation carriers, the average age of death was 66.6. In comparison, the life expectancy of individuals in the US in 2006 was 75.1 and 80.2 for men and women, respectively[23]. For most of the *TERT* mutation carriers, the cause of death was related to respiratory insufficiency. On average, the mean life expectancy of *TERT* mutation carriers with pulmonary fibrosis was 3 years from the time of diagnosis (**Figure 4B**). We find that a heterozygous *TERT* mutation status predicted a clinical outcome of progressive pulmonary fibrosis that mirrors the clinical course of IPF.

## Discussion

Genetic mutations in the *TERT* gene are the most frequent molecular defect found in patients with autosomal dominant pulmonary fibrosis. This disease can be considered a “telomeropathy,” like DKC, bone marrow failure and liver disease, when caused by germline mutations in telomerase and characterized by short telomere lengths. In this study, we describe the spectrum of diseases associated with heterozygous *TERT* mutations. This study was biased toward collecting subjects with pulmonary disease as the probands of each family were all collected based upon their known diagnosis of pulmonary fibrosis. However, as we expanded the kindreds, we discovered many other *TERT* mutation carriers with a similar phenotype. Overall, 40% of 134 *TERT* mutation carriers with a mean age of 51 carry a diagnosis of pulmonary fibrosis. All *TERT* mutations were discovered in individuals of white or Hispanic ancestry, similar to previous reports[18], [19].

This study demonstrates several similarities and differences between the different “telomeropathies.” First, the cardinal features of DKC, reticulated hyperpigmented skin, oral mucosal leukoplakia and dystrophic nails, are not seen in this cohort of *TERT* mutation carriers. Second, the age at the onset of disease can be very different. DKC is a usually a disease of childhood; bone marrow failure due to *TERT* mutations can affect individuals of a wide range of ages[24]. In contrast, the pulmonary fibrosis phenotype is age-dependent. None of the *TERT* mutation carriers less than 40 years of age have pulmonary fibrosis, but 60% and 50% of men and women, respectively, have pulmonary fibrosis at ≥60 years of age. Third, the clinical spectrum of disease seen in these *TERT* mutation carriers is milder than seen with DKC. Over 85% of DKC patients have a manifestation of bone marrow failure, 20% have pulmonary disease, and fewer have premature graying, extensive dental loss, esophageal strictures, peptic ulceration and an increased cancer predisposition[25]. Here we find aplastic anemia in 2 *TERT* mutation carriers, but isolated anemia in 18 individuals. Similarly, crytogenic liver cirrhosis is found in one *TERT* mutation carrier, but elevated liver function tests are seen in 11 individuals. Three other individuals for whom genomic DNA was not available for sequence analysis died from hepatitis-related liver cirrhosis. This is consistent with a recent report of a wide spectrum of familial liver disease in kindreds with telomerase mutations, including two *TERT* mutation families [26]. While bone marrow dysfunction or liver cirrhosis can be found concurrently with pulmonary fibrosis, these diseases are frequently found in different individuals. The compilation of diseases related to telomerase dysfunction seen in different members of the same family rather than in the same individual is a common feature of the *TERT* kindreds. The observations in these families suggest that the penetrance of the *TERT* mutation is incomplete, though substantial (∼40%) in causing pulmonary fibrosis and that expression is highly variable.

The “telomeropathies” are related to each other due to a shared pathogenic mechanism of telomere shortening. While telomere lengths are short in this *TERT* cohort, they are not as short as is usually seen with DKC patients. For the *TERT* mutation carriers in this study, 79% have telomere lengths shorter than the 10^th^ percentile and 56% have lengths shorter than the 1^st^ percentile of healthy controls. In contrast, 100% of DKC patients have lengths below the 1^st^ percentile of normals[27]. We see an age-dependent decline in telomere length for the *TERT* mutation carriers. In contrast, an age-dependent decline is not seen in DKC patients perhaps because the affected individuals may have already reached their minimal telomere lengths[28], [29].

Autosomal dominant DKC due to mutations in *TERC*, which encodes hTR, or *TERT* can demonstrate disease anticipation with increasing severity and an earlier onset in successive generations [10], [30]. The anticipation is due to inheritance of both shorter telomeres and the parental telomerase mutation. Here we show that although disease or death can occur earlier in successive generations, anticipation is not the rule for all the *TERT* kindreds. Given the lethality of pulmonary fibrosis and the late onset of this disease, DNA samples from individuals belonging to preceding generations were not available. However, evaluation of telomere lengths of DNA samples of following generations of *TERT* mutations has shown a statistical trend toward further telomere shortening for three large kindreds. Transmission of the *TERT* mutation from the father, rather than the mother, led to shorter mean telomere lengths of the children. Whether these short telomere lengths are due to the number of cell divisions of the spermatogonia, relative to the egg, prior to fertilization or to sex-related genomic imprinting is not clear. However, this finding is similar to other reports of a strong paternal influence on telomere length[31], [32].

We see shortening of telomere lengths for family members who are wild-type for both *TERT* alleles in comparison with two reference groups: healthy unrelated controls and spouses marrying into the family. These family members have evidence of telomere shortening in the absence of a *TERT* mutation; their telomere shortening represents an epigenetic modification that has been stably inherited from the parent with a *TERT* mutation. Inherited short telomere lengths in the absence of telomerase mutations have been reported previously in one large *TERC* kindred[28] and have been elegantly studied in mice. Genetic breeding strategies have produced mice with short telomere lengths; these exhibit degenerative defects even though telomerase is wildtype[33], [34]. We have previously reported that ∼25% of human subjects with the sporadic idiopathic interstitial pneumonias have short telomere lengths (<10^th^ percentile) in the absence of telomerase mutations[19], suggesting a role of this epigenetic modification in the development of non-familial pulmonary fibrosis. It is currently unknown whether human family members in these *TERT* kindreds have any clinical phenotypes that may be associated with short telomere lengths in the absence of an inherited telomerase mutation.

A major limitation of this study is that it is a family-based observational study. Due to ascertainment bias, the prevalence of pulmonary fibrosis is likely much higher for this group than a randomly collected population-based cohort with telomerase mutations. Although the questionnaires were completed prospectively by subject participants, medical records and studies were reviewed retrospectively. Not all subjects underwent the same work up for each medical diagnosis. Pulmonary fibrosis is an age-related phenotype and relevant clinical data was missing for many of the historical cases. CT scans of the chest were not widely performed prior to 1980. In addition, pathology review was not possible for all since many of the affected individuals had not undergone surgical lung biopsies. The exposure data is also fraught with recollection bias; those with lung disease may be more likely to remember the occupational or environmental exposures that they later have been told are associated with pulmonary fibrosis. It is also difficult to retrospectively quantitate past life-long respiratory exposures or obtain accurate exposure histories from deceased individuals. Despite these limitations, the pulmonary phenotype was characterized with available data.

Most of the originally reported *TERT* mutation cases had IPF, but some did not fit the narrow diagnostic criteria for this disease[18], [19]. One goal was to determine what percent of *TERT* mutation carriers represent IPF using modern diagnostic criteria[15]. The interstitial lung disease associated with the *TERT* mutations is characterized uniformly by dyspnea and a decreased diffusion capacity. Almost three-fourths have a radiographic pattern of pulmonary fibrosis that is typical of IPF. However, 13% have a pattern that is atypical for IPF, either with reticulation that is upper or mid-lung zone predominant or fibrosis that occurs along bronchi. An upper lung predominant pattern of fibrosis can be consistent with clinical diagnoses of sarcoidosis, pneumoconioses or chronic hypersensitivity pneumonitis, diagnoses which some of these individuals carried. However, despite a wide range of treatment courses, pulmonary fibrosis was progressive with a mean survival of 3 years from diagnosis. There seemed to be no difference in the radiologic and pathologic patterns related to specific *TERT* mutations.

The coexistence of inflammation and fibrosis may be a characteristic of organ disease associated with telomerase mutations. Over 35% of the chest CT scans showed enlarged mediastinal lymph nodes, a radiographic finding that is usually not seen with IPF. While UIP was the predominant pathologic feature in surgical lung specimens, 17% also had scattered histiocytes and/or non-necrotizing granulomas and 35% had increased amounts of interstitial inflammation. These features seen in the absence of UIP generally portend a better response to immunosuppressant medication. Liver disease associated with telomerase mutations is heterogeneous in severity and pathology with findings of co-existent fibrosis and inflammation[26].

Over ninety-five percent of the *TERT* mutation carriers have smoked or had an exposure to a fibrogenic agent that has been linked to the development of pulmonary fibrosis. This suggests a role of environmental factors in triggering lung injury in a tissue that is more susceptible. Some of the variable expressivity of the clinical phenotype of *TERT* mutation carriers may be related to environmental injury of susceptible cells and organs, with possible influences from other genetic and epigenetic factors. Since there is reduced penetrance of lung fibrosis even for the oldest age bracket, modification of environmental exposures may prevent or delay the onset of disease for those who have inherited this genetic risk. In the upcoming era of genomic medicine, it will become imperative to counsel the next generations of individuals with germline telomerase mutations to avoid exposure to any agents that can harm those cells and organs that are especially sensitive to telomerase dysfunction.

## Supporting Information

Figure S1 Evaluation of novel rare *TERT* mutations. (A) Sequence electropherograms of PCR products amplified from genomic DNA of individuals heterozygous for mutations in *TERT*. Wild-type (wt) and mutant cDNA sequences are listed directly below the tracings. Heterozygous missense mutations are indicated at the positions marked by the short arrows. (B) Amino acid alignment of the *TERT* sequences of *Homo sapiens* (human), *Macaca mulatta* (monkey), *Canis familiaris* (dog), *Bos taurus* (cow), *Mus musculus* (mouse), *Rattus norvegicus* (rat), *Gallus gallus* (chicken), *Xenopus laevis* (frog), *Schizosaccharomyces pombe* (yeast), and *Arabidopsis thaliana* (plant). (C) Relative telomerase activity of *TERT* mutations as measured by the telomere repeat amplification protocol (TRAP) assay are calculated as a ratio of the intensity of the sample's telomerase products to that of an internal control band and normalized to wild-type activity. Error bars represent the SD of duplicate experiments. Parallel reactions using [^35^S]methionine were run on a sodium dodecyl sulfate-polyacrylamide gel to confirm equal expression of the TERT wild-type and mutant proteins.
(1.16 MB TIF)

Figure S2 Correlation between telomere lengths measured by Southern blot (TRFL, kb) and by multiplexed real-time PCR (Relative LN(T/S ratio)) for 387 different genomic samples. The Southern blot method for determining telomere length (Terminal Restriction Fragment Length Analysis) was performed as described [19]. A relative LN (T/S ratio)  = 1 corresponds to a terminal restriction fragment length of 4.5 kb. By linear regression analysis, the correlation between the two are highly significant (Spearman's rank correlation  = 0.83, P-value <2.2×10^−16^).
(2.77 MB TIF)

Table S1 Distribution of *TERT* mutations for 134 heterozygous mutation carriers.
(0.01 MB PDF)
