# Supplementary figures and images for: Telomere Lengths, Pulmonary Fibrosis and Telomerase (TERT) Mutations
Source: PLoS One. 2010 May 19;5(5):e10680. doi: 10.1371/journal.pone.0010680 (PMC2873288; doi:10.1371/journal.pone.0010680)

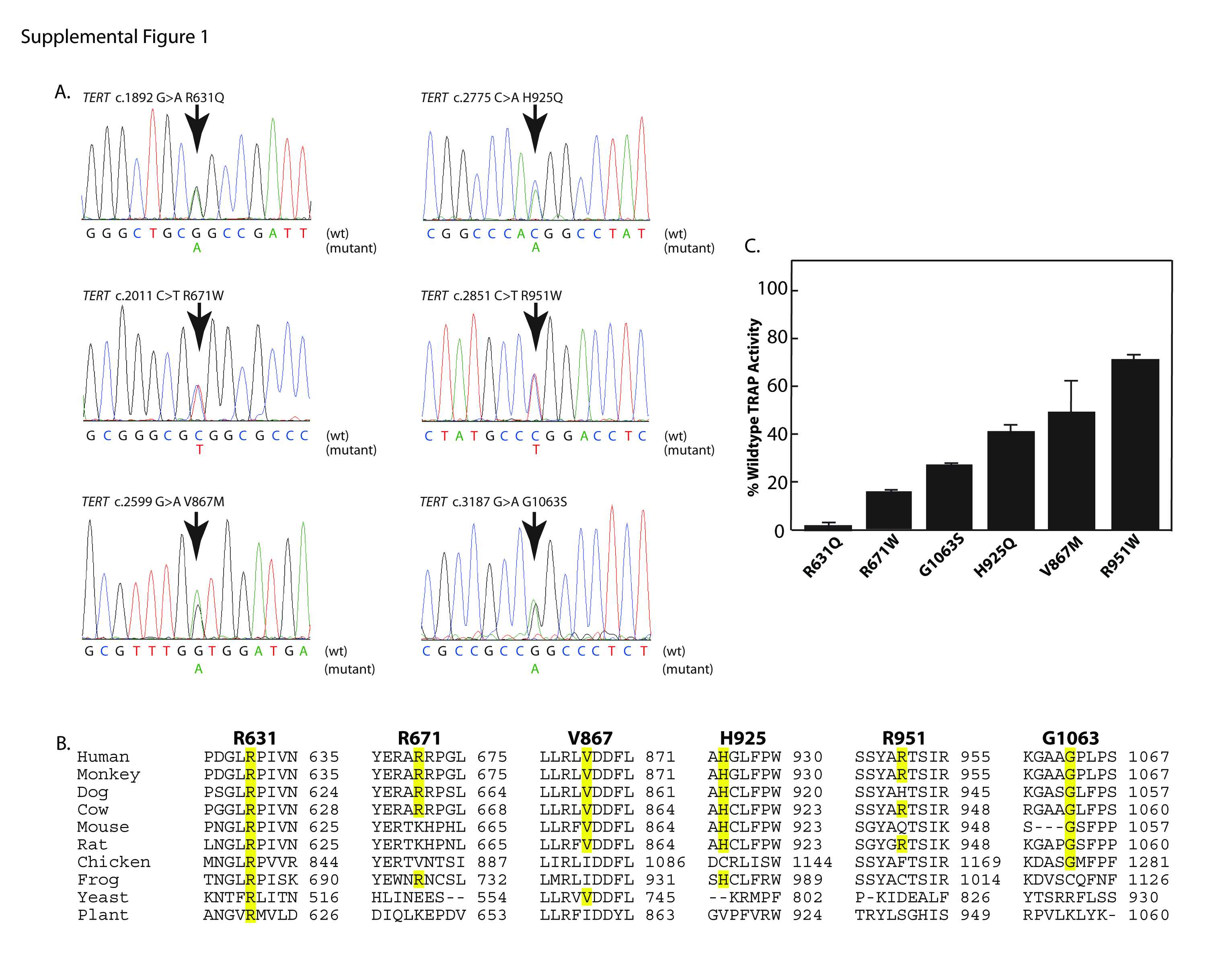

Supplement: Figure S1 — Evaluation of novel rare TERT mutations. (A) Sequence electropherograms of PCR products amplified from genomic DNA of individuals heterozygous for mutations in TERT. Wild-type (wt) and mutant cDNA sequences are listed directly below the tracings. Heterozygous missense mutations are indicated at the positions marked by the short arrows. (B) Amino acid alignment of the TERT sequences of Homo sapiens (human), Macaca mulatta (monkey), Canis familiaris (dog), Bos taurus (cow), Mus musculus (mouse), Rattus norvegicus (rat), Gallus gallus (chicken), Xenopus laevis (frog), Schizosaccharomyces pombe (yeast), and Arabidopsis thaliana (plant). (C) Relative telomerase activity of TERT mutations as measured by the telomere repeat amplification protocol (TRAP) assay are calculated as a ratio of the intensity of the sample's telomerase products to that of an internal control band and normalized to wild-type activity. Error bars represent the SD of duplicate experiments. Parallel reactions using [35S]methionine were run on a sodium dodecyl sulfate-polyacrylamide gel to confirm equal expression of the TERT wild-type and mutant proteins. (1.16 MB TIF) [file pone.0010680.s001.tif]

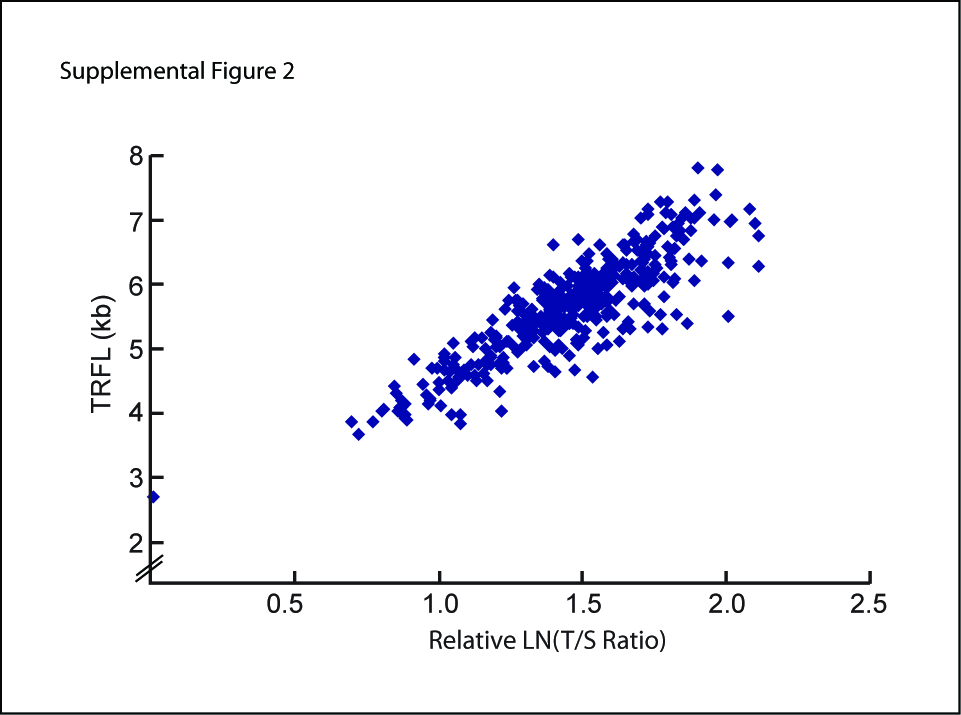

Supplement: Figure S2 — Correlation between telomere lengths measured by Southern blot (TRFL, kb) and by multiplexed real-time PCR (Relative LN(T/S ratio)) for 387 different genomic samples. The Southern blot method for determining telomere length (Terminal Restriction Fragment Length Analysis) was performed as described [19]. A relative LN (T/S ratio) = 1 corresponds to a terminal restriction fragment length of 4.5 kb. By linear regression analysis, the correlation between the two are highly significant (Spearman's rank correlation = 0.83, P-value <2.2×10^−16^). (2.77 MB TIF) [file pone.0010680.s002.tif]
