# Supplementary material for: Telomere Lengths, Pulmonary Fibrosis and Telomerase (TERT) Mutations
Source: PLoS One. 2010 May 19;5(5):e10680. doi: 10.1371/journal.pone.0010680 (PMC2873288; doi:10.1371/journal.pone.0010680)
Supplement: Table S1 — Distribution of TERT mutations for 134 heterozygous mutation carriers. (0.01 MB PDF) [file pone.0010680.s003.pdf]

Supplemental Table 1. Distribution of *TERT* Mutations for 134 Heterozygous Mutation Carriers

Mutation	Number of Subjects	Percent (%)
P33S	3	2.2
V144M	30	22.4
R486C	6	4.5
R631Q	6	4.5
R671W	1	0.7
V694M	2	1.5
P702L	24	17.9
P704S	4	3.0
V747fs	12	9.0
R865H	18	13.4
R865C	2	1.5
V867M	1	0.7
H925Q	2	1.5
R951W	5	3.7
S957R	3	2.2
L1019F	2	1.5
K1050E	2	1.5
G1063S	5	3.7
E1116fs	4	3.0
Total	134	100
